# Early social-cognitive development as a dynamic developmental system—a lifeworld approach

**DOI:** 10.3389/fpsyg.2024.1399903

**Published:** 2024-06-13

**Authors:** Joscha Kärtner, Moritz Köster

**Affiliations:** ^1^Developmental Psychology Lab, Department of Psychology, University of Münster, Münster, Germany; ^2^Developmental Cognitive Psychology, Department of Psychology, University of Regensburg, Regensburg, Germany

**Keywords:** social-cognitive development, developmental systems, dynamic systems, lifeworld approach, self-awareness, helping, social learning

## Abstract

Based on developmental systems and dynamic systems theories, we propose the lifeworld approach—a conceptual framework for research and a hypothesis concerning early social-cognitive development. As a framework, the lifeworld approach recognizes the social embeddedness of development and shifts the focus away from individual developmental outcomes toward the reciprocal interplay of processes within and between individuals that co-constitutes early social-cognitive development. As a hypothesis, the lifeworld approach proposes that the changing developmental system—spanning the different individuals as their subsystems—strives toward attractor states through regulation at the behavioral level, which results in both the emergence and further differentiation of developmental attainments. The lifeworld approach—as a framework and a hypothesis, including key methodological approaches to test it—is exemplified by research on infants' self-awareness, prosocial behavior and social learning. Equipped with, first, a conceptual framework grounded in a modern view on development and, second, a growing suite of methodological approaches, developmental science can advance by analyzing the mutually influential relations between intra-individual and interactional processes in order to identify key mechanisms underlying early social-cognitive development.

## 1 Early social-cognitive development as a dynamic developmental system

It is a mantra in the developmental sciences that nature and nurture interact in complex ways in human development. For instance, developmental systems approaches (e.g., Oyama, 2000; Gottlieb, 2007; Overton, 2013) conceive ontogeny as the dynamic interplay of internal and external factors at different levels (e.g., genes, brain, behavior, ecology, culture). Relatedly, transactional theories emphasize that throughout ontogeny there exists a dialectic interplay of nature and nurture (e.g., Sameroff, 2009).

Yet, many contemporary theories in developmental psychology focus on the development of the individual without taking into full account that social-cognitive development is deeply embedded in rich and versatile social contexts (e.g., Tomasello et al., 2005; Csibra and Gergely, 2009; Warneken and Tomasello, 2009). As such, even if these theories acknowledge the influence of social interaction (e.g., in the sense that there must be a model to imitate, a partner to collaborate with or a group to belong to), they largely underestimate the role that the context and others play in co-constituting early social-cognitive development. While the concept of co-regulation through others and its effects on child development is a key concept for the field of social-emotional development [see, for instance, attachment theory (Bowlby, 1969) or work edited by Cole and Hollenstein (2018)], we argue that it is just as essential to systematically integrate interpersonal components of the developmental system in the domain of social cognition in order to fully understand early development. To address this gap in the field of social-cognitive development, it is essential to analyze the bidirectional and changing relationships between the experience and behavior of the child and the experience and behavior of others in the close social network. These bidirectional interactions, as part of the developmental system, need to be considered more profoundly in theory and research in order to adequately describe social-cognitive development and its driving mechanisms and forces.

**Figure 1:**
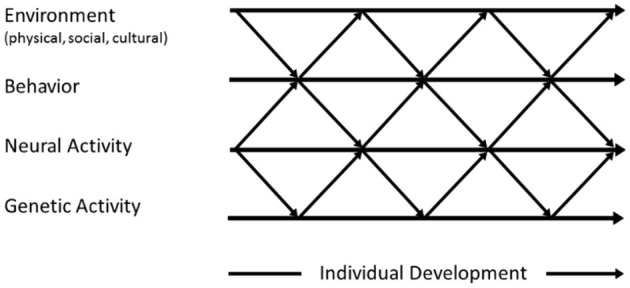
The basic idea of the developmental systems account of the ontogeny of organisms, conceiving individual development as an interplay between genetic, neural, behavioral, and environmental processes.

We start from the basic assumption that human ontogeny is best understood as a developmental system (Oyama, 2000; Gottlieb, 2007). In his probabilistic epigenesist framework, Gottlieb emphasizes that the development of all organisms is the result of a dynamic interplay between genetic activity, neural activity, behavior and the influence of the physical, social, and cultural environment, with a close interdependency between those levels (see Figure 1). According to this perspective, development always and in all domains is the result of the regulation between organismic and experiential factors, with neither type having priority over the other. As such, the “…notion that phenotypic traits, including behavior, can be predetermined has slowly given way in biology and psychology… [due to]… growing evidence for the fundamental role of developmental processes in the generation of the stability and variations in phenotype.” (Gottlieb, 2007, p.1).

Gottlieb's theory is mainly grounded in animal research and is mute on psychological processes—such as motives and cognition—associated with neural activity, behavior or factors external to the individual. Here, we join others (e.g., Sameroff, 2009; Szokolszky and Read, 2018) and extend the basic tenets of the developmental systems approach to psychological processes, specifically the motivational and cognitive processes, which are ubiquitous in theories on children's social-cognitive development. In doing so, the lifeworld approach focuses on the reciprocal relationship between these psychological processes and key external factors. Specifically, we focus on regulation processes occurring within social interaction that are situated within the broader socio-cultural context. These are, in our view, essential to understand early development also in the social-cognitive domain and may best be understood from a developmental systems perspective that integrates further concepts of dynamic systems theories, which we turn to next.

Drawing on dynamic system theories, we propose that the reciprocal co-regulation of the internal and external forces that constitute the developmental system can best be characterized as a complex and non-deterministic process. In this view, “development can only be understood as the multiple, mutual, and continuous interaction of all the levels of a developing system, from the molecular to the cultural” (Thelen and Smith, 2007, p. 258). A critical property of a dynamic system is that it settles into only a few stable configurations, so called attractor states (van Geert, 1994; Van Geert and Van Dijk, 2002; Thelen and Smith, 2007). That is, out of the many potential states of a system, it is a limited number of states that occur as recurrent stable forms. Importantly, these attractor states are not static but are often described as forms with dynamic stability. This means that, in any given situation and depending on the developmental status of the individual, there is ongoing interaction and reciprocal regulation around one or more attractor states and, across development, the attractor states may change [see also Waddington's (1957) epigenetic landscapes].

**Figure 2:**
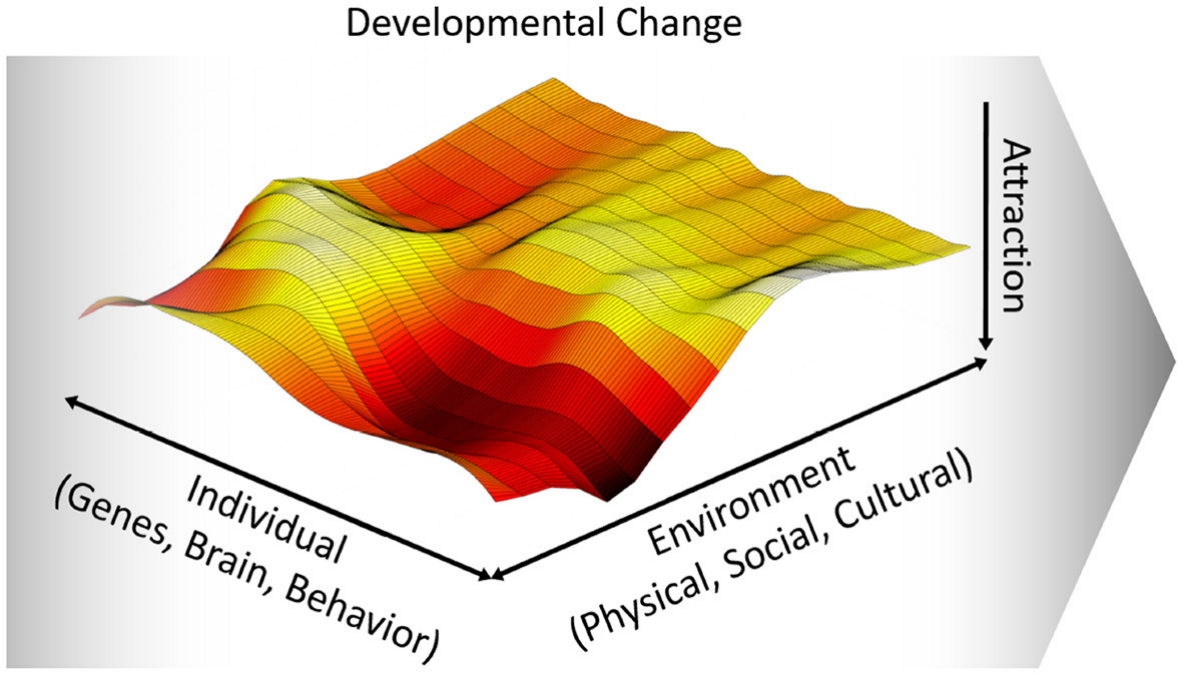
Visualization of the developmental landscape with attractor states. The dynamic developmental system depends on the current state of individual and environmental factors. Here we conceptualize the dynamic developmental system as a field of forces that drives early (socio-cognitive) development over time.

The resulting dynamic developmental system can be conceptualized as a field of forces between individual (genes, brain, behavior) and external (physical, social, cultural) processes that span a developmental landscape (Figure 2), which changes dynamically over development. Attractor states within this landscape can be defined as potential developmental outcomes, which are stabilized patterns of interacting with the environment. For a more general example, let us consider a toddler learning to climb stairs from a dynamic developmental system perspective. There may be different strategies which change dynamically over time, from crawling, to taking stairs step by step with both feet, to climbing stairs confidentially, taking one stair with one foot at a time. This may be modeled, trained and scaffolded by others, shaping the learning process in concert with infants' growing ability and confidence. Different strategies to climb stairs can be conceptualized as attractor states, which change over time with infants' growing physical experience and in interaction with the physical environment (i.e., the stairs) and social influences (i.e., modeling, training and social support by others). In the following, we shift our focus and elaborate a dynamic developmental systems perspective for early social-cognitive development. This implies that—at any given time-point—socio-cognitive development strives toward stable patterns of experience and behavior that are informed by the developmental status and individual features of the child and others' co-regulation.

Based on these fundamental assumptions on early development, we now introduce the lifeworld approach as a theoretical framework for describing and explaining early social-cognitive development. We will then ground the lifeworld perspective in empirical evidence from three key domains of early social-cognitive development, namely self-awareness, prosocial behavior and social learning, before we elaborate critical implications for theory and future research.

## 2 A lifeworld approach to social- cognitive development

The lifeworld approach is based on the assumption—shared with many other developmental approaches—that the experiences children have when interacting with their social environment are essential to the understanding of early child development (e.g., Vygotsky, 1997; Rogoff, 2003; Keller, 2007; Keller and Kärtner, 2013). In the approach suggested here, building on the dynamic developmental systems approach outlined above, the lifeworld is defined as the entirety of forces that constitute children's experience and behavior in a given situation, which in turn drive their development over time. First, there are the internal forces, namely the current motivational and cognitive processes that are linked to children's behavior at a given timepoint. Second, these internal forces are complemented by external forces in the social environment. As in Schütz and Luckmann's (1973) conception of the lifeworld, one of the key goals here is to trace the taken-for-granted and the common ground that characterizes individual experience and behavior in everyday life. In their approach, the lifeworld is socially constituted and conceptualized as a cultural and historical construction that turns “natural things into cultural objects (and) human bodies into fellow-men” (p. 5). Extending the original concept, the framework proposed here adds important internal biases and focuses on the dynamic interplay of the internal and external forces that, in concert, constitute everyday experience and behavior. Most importantly, both these forces directly influence children's experience and behavior by channeling attention to relevant information, both outside and within the children's organism and by providing blueprints and models of potential modes of experience and behavior that characterize everyday experience and behavior. These two interrelated forces are of primary importance for channeling the information that is available to children and that form children's experience and behavior in a given situation. These forces are embedded in larger and related timescales, with important consequences for children's learning and development. Together, these internal and external forces powerfully structure children's experiences and behavior and, over time, shape children's social-cognitive development.

**Figure 3:**
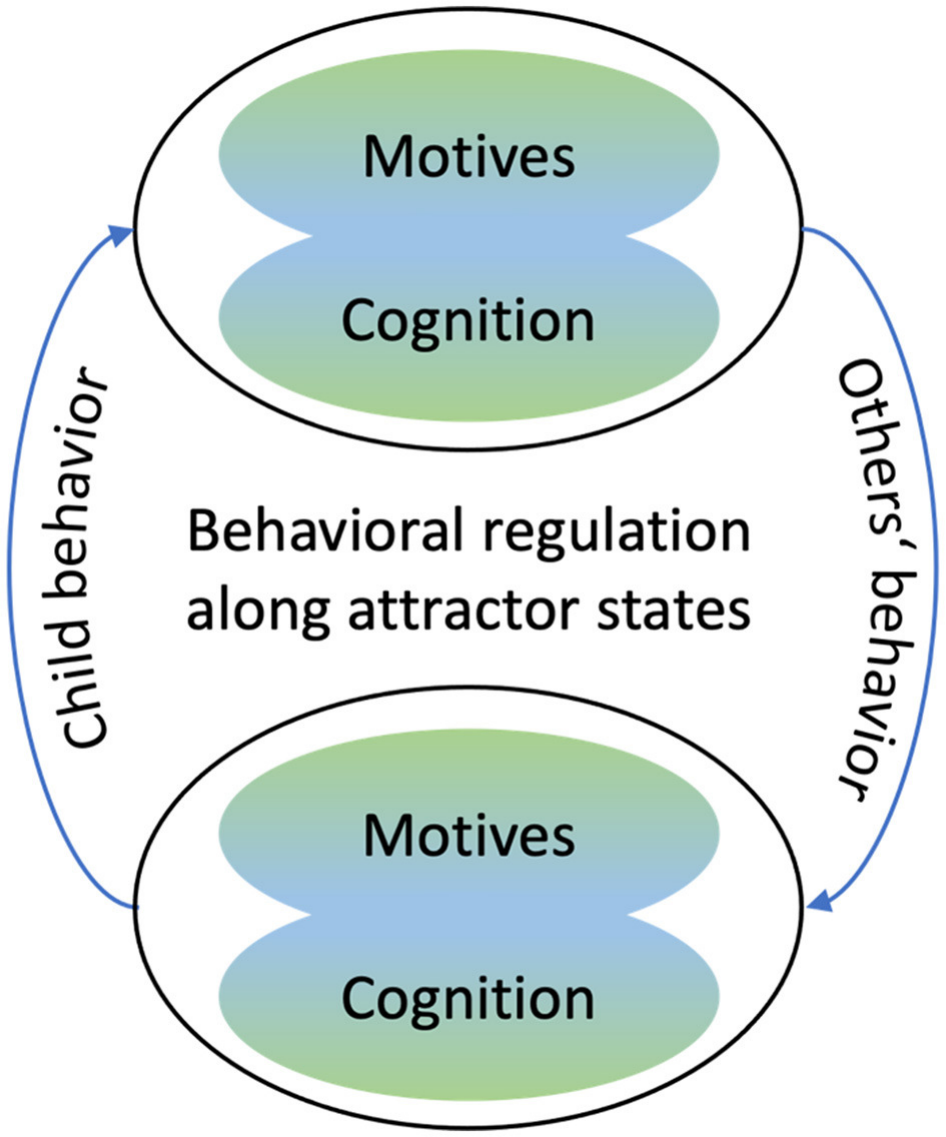
The dynamic developmental system spanning the child and others (in this case a dyadic context) and their reciprocal relations at the behavioral level, illustrating the core elements of the lifeworld approach.

Importantly, not only the child, but each individual of the dynamic developmental system can be conceptualized as a subsystem that, at any given moment, can be characterized by its own motives and cognitive states. In that sense, the lifeworld approach goes beyond considering others' influences on the individual as a further force within a dynamic system that is located within the individual and conceptualizes both child and others as integral constituents of the dynamic developmental system. As a consequence, the dynamic developmental system spans the child and other individuals, with each individual having their motives and cognition that drive individual behavior that reciprocally interact at the behavioral level. More specifically, the different subsystems are linked through verbal and nonverbal behavior, including attention. Thus, it is on the behavioral level that the internal and external forces of the subsystems interact. Through social interaction, the individuals influence each other's behavior and, in consequence, each other's cognition and motives. Through this mechanism, namely the behavioral co-regulation via social interaction, the internal forces of one individual become the external forces on another individual (see Figure 3).

Thus, in the framework suggested here, Gottlieb's principle of the reciprocal interplay of internal and external factors at different levels translates into the cognition and motives of individuals who reciprocally interact with each other at the behavioral level. More specifically, and as we will illustrate with different examples further below, the child's behavior at any given timepoint is informed by their current motives and cognition and will be interpreted and responded to by others in the light of their current motives and cognitive states. The others' response, in turn, may lead—via changes at the behavioral level—to changes in children's cognition and motives.

We here illustrate this basic idea by two examples from language development and prosocial behavior, before we elaborate the concept of the lifeworld approach in depth in the following section. In early vocal learning, when infants begin to make more mature vowel-like sounds around 4 months, caregivers are more likely to respond contingently to these more mature sounds, which leads to a pruning of children's vocal repertoire on an ontogenetic timescale (Venditti et al., 2023). Along these lines, experimental studies have shown that mature sounds occur more often and integrate the phonological patterns of their caregivers' speech when responded to contingently (Goldstein and Schwade, 2008). Venditti et al. further argue that, as infants' vocal learning progresses, caregivers update their expectations for vocal maturity (i.e., their cognition concerning to the lifeworld approach), which informs the probability of contingent responses to infants' vocal sounds, constituting a social feedback loop in a co-developing social system. Concerning early helping in the second year, a cross-cultural study on early helping has shown that depending on whether children's helping is conceptualized as a personal choice vs. an interpersonal obligation (maternal cognition), mothers differ whether they request something from their child by asking, pleading and giving explanations vs. by serious and insistent requests, which has implications for children's prosocial motivation, namely the degree to which they help based on a sense of interpersonal responsibility for shared chores (Köster et al., 2016a). Importantly to the lifeworld approach, it is through these bidirectional social interactional processes that the developmental system changes and, hence, the child develops. That is, either new developmental attainments emerge (including motives and cognition), or they become further differentiated at increasingly complex and novel levels of organization.

As a consequence, a child's current motives and cognitive processes are not conceptualized as “given” or “innate” but are the result of a biologically prepared and reliably emerging repertoire of internal biases and behavioral inclinations that, if integrated in social interaction, stabilize and manifest in habitualized reciprocal transactions between intra- and interpersonal processes up to this point in development. That is, others play an important role for the emergence and further differentiation of social-cognitive developmental attainments by coherently organizing children's behavior and, as a consequence, children's developing motives and cognition: By their structuring, others guide children's attention, they prompt desirable behavior, and they correct or discourage undesirable behavior during everyday activities (Kärtner, 2018). In sum, the lifeworld approach focuses on the internal forces (i.e., cognition and motives) that inform the subsystem's (i.e., the child's and others') behavior plus the reciprocal co-regulation between the subsystems at the behavioral level. Narrowing the focus down to the child, these are the internal and external forces that drive a child's experience and behavior and, in consequence, their development.

Considering the diversity of human cultures, the lifeworld approach also offers a conceptual framework to account for potentially culture-specific development. More specifically, by following specific cultural models—defined as shared systems of meaning associated with ideal child development and optimal caregiving that vary across populations and that have normative force (Keller and Kärtner, 2013)—caregivers may follow different agendas. These agendas set different target states for the developmental system, which has direct implications for their motives and cognition when providing and structuring settings for their children (Kärtner, 2018). In the next sections, we will review evidence that shows that—depending on the cultural model's ideal target states—children's behavior may be interpreted and reacted to differently, which has important implications for children's development. Along these lines, cross-cultural studies help to illustrate systematic differences in children's external forces (i.e., others' cultural models), leading to culture-specific attractor states that may emerge within an individual's developmental landscape.

Overall, the lifeworld approach to social-cognitive development considers behavioral co-regulation toward stable attractor states of increasing complexity as a key mechanism underlying early social-cognitive development. We derive from this that it is essential to describe not only children's but also caregivers' experience and behavior and their reciprocal relationship. Doing so requires a process perspective that focuses on the dynamics of social interaction that enables researchers to identify the interactional mechanisms that help explain early social-cognitive development and its inter-individual and cross-cultural variation. In the following, we review studies from three different areas within social-cognitive development that illustrate the key assumptions and that provide convergent evidence for central claims of the lifeworld approach. At the same time, we sketch different methodological approaches that allow to address social-cognitive development from a dynamic developmental systems perspective.

### 2.1 The emergence of self-awareness during the first months of life

In the first months of life, infants' self-awareness emerges within social interaction with their primary caregivers. One proximal mechanism that strengthens infants' sense of agency and sensitizes infants to their internal mental states has been suggested by Gergely and Watson (1996, 1999) in their social biofeedback model. The authors' basic idea is that—grounded in infants' interest in human faces and their sensitivity for contingent responsiveness—infants are sensitized to their own internal states by caregivers' repeated displays of partially imitative and marked reflections of infants' affective and intentional states.

One developmental indicator of increasing self-awareness is the 2-month shift that describes a qualitative change in the way infants interact with their social environment: they become more attentive, look longer at others' faces, and start smiling socially during contingent social interaction. Based on findings from longitudinal studies, Lavelli and Fogel (2002, 2005) describe the mother-infant dyad as a dynamic, co-regulatory system that stabilizes around specific attractor states, namely the visually attentive and positively aroused infant. More specifically, by combining ontogenetic with dynamic interactional analyses, the study by Lavelli and Fogel (2005) shows that, across the first months of life, certain behaviors of the mother and infant elicit specific reactions from the interaction partner, leading to cycles of mutual amplification. For instance, while maternal talk or smile is both contingent on and leads to infants' simple attention in the first month, there are strong bidirectional links between maternal talk or smile and both infant cooing and smiling in the third month of life. Thus, with infant age, these patterns become increasingly more complex and reliable and, therefore, the developmental system establishes and stabilizes the behavioral patterns associated with the 2-month shift that is indicative of infants' emerging self-awareness.

While this co-constructive theory (see also Messinger and Fogel, 2007) assumes that the developmental system—in this case consisting of the developing infant and the mother—stabilizes around a universal ideal state, namely the visually attentive and positively aroused infant, other studies have shown that these ideal states may differ considerably between cultures (Wefers et al., 2022). More specifically, ideal states of infant behavior are part of caregivers' cultural models, defined as their shared beliefs about infant development and associated practices. If the cultural model is about positive emotionality within face-to-face interaction, caregivers will be motivated to establish and sustain mutual gaze and social smiling in their infants (Kärtner et al., 2013; see also Kärtner, 2015). However, if the cultural model is about the quiet and calm child, social smiles may pass by unnoticed. Along these lines, cross-cultural studies have shown that the development of both mutual gaze and social smiling are contingent on cultural beliefs (i.e., caregivers' cognitive states that have implication for their motives when interacting with their infants) and practices: Only if caregivers value mutual gaze and social smiling—as indexed by culture-specific ethnotheories on ideal infant affect (Wefers et al., 2022), contingency patterns (Kärtner et al., 2008, 2010) or affect mirroring (Wörmann et al., 2012, 2014; Broesch et al., 2016)—will their infants show an increase in mutual gaze and smiling—an indicator of accentuated self-awareness—toward the end of the second month (Kärtner et al., 2010, 2022; Wörmann et al., 2012, 2014).

Overall, these studies—that use different methodological approaches, specifically longitudinal observations, sequential analyses and cross-cultural comparisons—converge on the conclusion that infants' self-awareness emerges within the dynamics of the developmental system spanning the infant and their primary caregivers who stabilize the developmental system along the target states implicated in their cultural models of optimal caregiving and ideal child development.

### 2.2 Prosocial behavior—the development of early helping

In line with dynamic developmental systems theory, Kärtner and Köster have argued that the emergence and further differentiation of helping (i.e., behavior that supports others to achieve an instrumental goal) during the first 2 years of life is co-constituted in social interaction (Köster et al., 2015; Kärtner, 2018; Köster and Kärtner, 2019). Developmental research, based largely on experimental studies in Europe, the United States, and Japan, suggests that basic elements of prosociality—social and prosocial cognition and motivation and prosocial behavior—emerge during the first and second year of life (see also Kärtner, 2018). Already 3- and 6-month-olds are capable of social evaluation, preferring characters that help (vs. hinder) others (Hamlin, 2013) and, from 9 months of age, infants understand others' needs and intentions, which build the necessary foundations for genuinely prosocial early helping (e.g., Woodward, 1998; Behne et al., 2005; Köster et al., 2016b, 2019). Both lab studies and ethnographic research has shown that young children around the world are highly motivated to engage in helping and cooperative activities, such as adults' chores, by pitching in proactively and on request (for a review, see Kärtner, 2023).

Concerning the occurrence of early helping, there is evidence that caregivers' structuring—especially encouragement and praise—early in the second year has both concurrent and longitudinal effects on toddlers' helping (Dahl, 2015; Hammond and Carpendale, 2015; Giner Torréns and Kärtner, 2019; Kärtner et al., 2021; Dahl et al., 2022). Furthermore, there is evidence showing that experimenters' encouragement and praise increases toddlers' helping when toddlers are 13 to 15 months old but not above that age (Dahl et al., 2017). The findings of this experimental study suggest that specific aspects of social interaction are effective influences and confer their co-constitutive forces only during specific periods in development. Furthermore, observing prosocial models has been shown to increase 16-month-olds' helping behavior (Schuhmacher et al., 2019). Finally, addressing this question from a cross-cultural perspective, a recent study has shown that 18-month-olds from Delhi, India, helped more often than toddlers from Münster, Germany (see also Callaghan et al., 2011; Giner Torréns and Kärtner, 2017).

The behavioral regulation along specific target states within the dynamic developmental system not only affects the frequency of helping, but similarly supports the differentiation of prosocial motives across development. In a recent review, Kärtner (2023) suggested a developmental progression in which the earliest form of prosocial motives, namely children's participation in joint endeavors rooted in a sense of belonging during toddlerhood is complemented by children's contributions based on their sense of responsibility in early childhood and their sense of normative obligation based on moral considerations during middle childhood. Cross-cultural studies by Miller and colleagues have shown that these moral considerations on helping differ across cultures. More specifically, helping is rather grounded in duty-based considerations in Hindu Indians and on more voluntaristic considerations in European Americans, where helping is seen as a matter of personal moral choice (for a review, see Miller et al., 2017).

Along these lines, there is empirical evidence that cultural models of helping affect caregivers' structuring, which has implications for the development of early helping (Köster et al., 2016a; Giner Torréns and Kärtner, 2017). Giner Torréns and Kärtner (2017) report that mothers from Delhi provide more opportunities for helping and express higher levels of disapproval if their children do not help them, while mothers from Münster reported more praise after helping. Furthermore, there were culture-specific associations between helping and disapproval that indicate that these culture-specific ways of structuring may contribute to the differentiation of early helping behavior, such as helping that is motivated by a self-determined personal choice vs. an interpersonal obligation. Further evidence for this idea comes from a cross-cultural study that demonstrates that mothers' structuring during chore assignment differs between cultures and has consequences for toddlers' prosocial behavior: while helping was associated with assertive structuring (i.e., serious and insistent requesting) in a rural Amazon region in Brazil, it was correlated with deliberate scaffolding (i.e., asking, pleading and giving explanations) in a Western urban middle-class context (Köster et al., 2016a). These findings further support the idea that others regulate the cognitive and motivational foundations of children's early helping behavior, along the lines of interpersonal responsibility and personal choice.

Overall, there is converging evidence from different methodological approaches, including cross-sectional and longitudinal correlational and experimental designs and cross-cultural studies showing that both the emergence and further differentiation of helping are a product of the relational developmental system and critically depend on behavioral regulation through others that is informed by caregivers' cultural models of children's helping. Overall, these studies converge on the conclusion that any theoretical account that ignores the constitutive role of social interaction for the emergence and differentiation of prosocial behavior is incomplete.

### 2.3 Social learning—aligning with others through cultural learning and normativity

Learning from others—acquiring behavioral repertoires from others through observation and instruction—allows young children to acquire the behavioral routines and norms of their culture from close others, which lays the foundation for humans' ability to adapt to a variety of social and ecological environments (Keller, 2007; Over and Carpenter, 2012; Shneidman and Woodward, 2016; Legare, 2017; Köster, 2024). From as early as 6 months of age, infants are capable and motivated to imitate simple actions (Meltzoff, 1988; Meltzoff and Moore, 1994; Barr et al., 1996). Cognitively, it has been shown that from around this age, infants associate imitative behaviors with group membership and, from around 8 months, infants further expect that the members of a group act alike (Powell and Spelke, 2013, 2018). Furthermore, there is recent evidence that already 11-month-olds grasp the normative force of behavior observed in others and that behaving like others forms the basis for social evaluation (Köster and Hepach, 2024). So how are these prerequisites for early social learning, as the basis for cultural learning and normativity, shaped in social interaction with others?

Based on children's inclination for social learning, there are huge differences what is learned from whom and how. Considering the important role of teaching, social learning is co-constructed within an individual's social and cultural environment and shapes the underlying motives and cognition in culture-specific ways (Rogoff, 2003; Keller, 2007; Heyes, 2018), which also may result in specific differences in learning from others across cultures (Callaghan et al., 2011).

Concerning teaching, there is evidence that teaching behavior toward infants differs profoundly across cultures (Rogoff et al., 1993; Keller, 2007; Little et al., 2016; Köster et al., 2022). For example, Little et al. (2016) found that, when demonstrating a novel action to their infants, US-American urban middle-class parents showed a specific target action more often than mothers from villages in Vanuatu. Furthermore, during joint mealtime interactions with their 2-year-olds, parents from urban Germany, Argentina, and Japan relied more on abstract communication, the provision of choices and demonstration, while parents from rural Ecuador and Brazil relied on prompting their children what to do (Köster et al., 2022). These differences in parental socialization have been characterized as the basis for more autonomous developmental pathways in educated, industrialized cultural context, and toward hierarchical relatedness in rural, subsistence-based communities (Greenfield et al., 2003; Keller, 2007) with consequences for the underlying motives and cognition for complying with others' behaviors (Keller and Kärtner, 2013; see findings from early prosocial development detailed above).

Evidence showing that early differences in teaching are linked to differences in infants' social learning comes from comparisons between infants from Yucatec Mayan and US-American families. It has been described that children in Yucatec Maya communities in Mexico are socialized to keenly observe others' activities, whereas learning in urban US-American contexts relies more on direct teaching (Rogoff et al., 1993; Rogoff, 2003). Across developmental time, these differences have implications for the differentiation of infants' learning capacities: In the US, 18-month-old infants learned much better from a child-directed instruction as compared to a third-party scenario, whereas Yucatec Mayan toddlers learned equally well across conditions (Shneidman et al., 2016). These findings correspond to the cross-cultural differences in the development of children's learning from others' activities, described later in development, characterized as guided participation in Mayan communities or assembly line instruction in US contexts (Rogoff et al., 2003). That is, in Mayan communities, infants are expected to learn by participating in daily activities, which shapes their spontaneous attention and engagement in others' activities, while in the US infants are thought to receive more explicit instructions, which shapes their attention and learning capacities to rely on more direct forms of guidance by others. From a dynamic developmental systems perspective, the subsystem of the child is proposed to strive toward the culture-specific structuring of learning settings.

Overall, critical evidence that early learning capacities are co-constituted in early social interactions mainly comes from cross-cultural studies. These lines of research characterize differences in caregivers' teaching and infants' learning from others that support the idea that early social learning capacities are co-constructed in early social interactions, shaping underlying motives and cognition associated with learning from others. While some prototypical patterns of caregivers' and children's behavior emerge in the two lines of cross-cultural research outlined above, future research should focus on longitudinal studies, considering both parental behaviors and children's early social learning pattern, at distinct developmental time-points. Specifically, studying within-cultural variations may be key to better understand the fine-tuned and co-regulatory nature of early social learning capacities. In future, this may allow us to better understand the dynamic system underlying differential pathways in learning from close others, as the basis for early cultural learning and acquiring social norms.

## 3 Implications of the lifeworld approach and future perspectives

The lifeworld approach is both a framework for research and a hypothesis. As a framework, it combines concepts from developmental systems and dynamic systems theories and recognizes that social-cognitive development is co-constituted by children's lifeworld that is defined by the internal and external forces that are reciprocally linked at the behavioral level. As a hypothesis, it proposes that the external forces, namely the structuring of children's experience through others during social interaction, is systematically linked to the emergence and further differentiation of new developmental attainments. In support of the approach as a hypothesis, we reviewed evidence from specific developmental phenomena from the fields of self-awareness, early prosocial behavior and social learning.

More specifically, the lifeworld approach highlights (i) that internal processes, namely the individuals' motives and cognition, inform individual behavior, (ii) that through social interaction the different subsystems (i.e., the different individuals) influence each other's behavior, and (iii) that each individual's behavior is directly linked to their experience, that is, each individual's motives and cognition. Thus, changes at any level (i.e., either children's or others' cognition, motives or patterns of social interaction) are associated with dynamically interacting changes at all other levels, leading to developmental change. The key objective of the lifeworld approach is to advance our understanding of development by emphasizing the internal and external forces that drive children's experiences and behavior and thereby co-constitute their social-cognitive development.

An important implication of such a perspective is the shift from individual social-cognitive developmental outcomes to the dynamic processes underlying change within the developmental system. In our view, it seems essential to take into account these complex relationships between intra- and interindividual processes in order to adequately characterize the field of forces that constitute child development. As a consequence, it becomes key to analyze how changes in children (e.g., the emergence of social smiling) leads to changes in caregivers (e.g., their marked reflections of infant smiles, if infant smiling is appreciated as the ideal infant affect by caregivers) that lead to further changes in children (e.g., cascading effects on infant smiling and infants' self-awareness). Similarly, parental motivation and cognition of why and how to learn from others (e.g., socialization toward autonomy or complying with others' expectations, or to learn by observation vs. instruction) may shape infants motivations and cognition (e.g., attending to information that is not directly directed at the child) of why and how to align with the behaviors of others, and to become competent and accepted members of their cultural group.

As a framework for research, the lifeworld approach calls us to empirically test if and to what degree the dynamic of the developmental system is driven by caregivers' cognition and motivations, for instance, their cultural model of optimal caregiving and ideal child behavior and development that defines the target states the dynamic system strives for. Following this approach allows to identify universally uniform, as well as inter-individually and cross-culturally variable aspects of child development and to unravel the mechanism leading to an individual's developmental pathway.

There is a suite of methodological approaches that allow to analyze the characteristics of the dynamic developmental system as a field of forces that drive early development. From a lifeworld perspective, the focus lies on features of the social interactions that contribute to the emergence or differentiation of a specific aspect of social-cognitive development. Most of these methodological approaches have been touched on above: first, it is an important first step to describe the age-graded changes in child behavior and the complementary behavior of caregivers in order to document the specific experiences that children have at certain ages (e.g., Keller, 2007). These age-graded changes hint at potential reciprocal relations between current structuring and developmental processes.

Second, a powerful tool is to analyze the associations between specific features of social interaction and developmental outcomes, typically based on interindividual differences in children's and caregivers' behavior. Of these, prospective associations between current caregivers' behavior and later child outcomes after controlling for current child behavior—either in the form of cross-lagged or residual change analyses—are powerful designs, especially when capturing everyday interaction in ecologically valid situations, to support the proposal that developmental change is contingent on social interaction (e.g., Dahl, 2015; Kärtner et al., 2021). Thus, to understand developmental change over time, longitudinal studies that capitalize on both, parental beliefs and practices (setting differential target states over development) in combination with developmental outcomes (being driven by these target states), are an efficient tool to better understand how co-regulatory dynamics drive early socio-cognitive development over time. Third, experimental and training studies have the potential to test whether specific aspects of social interaction have situational effects on child experience and behavior (in the case of experiments) or long-lasting effects on child development (in the case of training studies) at a specific age.

Furthermore, cross-cultural studies, as a quasi-experimental design, allow to test whether developmental outcomes differ across cultures, depending on differences in caregivers' cultural models that inform their cognition, motives and behavior. That is, caregivers' cultural models may set specific and testable target states that inform caregivers' appraisals, motives and behavior, which may lead to a culture-specific developmental pathways which are testable in children's behavior at distinct developmental timepoints. Cross-cultural designs are most powerful, when developmental outcomes are assessed in a standardized way, complemented by naturalistic observations of everyday interaction and assessment of caregivers' meaning systems (Kärtner et al., 2022; Wefers et al., 2022, 2023).

Finally, within-person or within-dyad designs are the method of choice if one is interested in dynamic change and how the subsystems, namely the children and their caregivers, are reciprocally related to each other in social interaction as it unfolds in time. Coming closest to the process perspective, microgenetic designs allow to analyze the reciprocal relationships between child and caregiver behavior, that is, how child and caregiver affect each other in real time. An exemplary study is the one from Lavelli and Fogel (2002) described above, that demonstrated, based on sequential analyses, that changes in infant smiling and cooing led to direct changes in maternal talking and smiling and vice versa, leading to cycles of mutual amplification, especially in the third month of life, suggesting a positive attractor state of the dynamic developmental system. Currently, methods for dynamic analyses are flourishing in the field of developmental science and there are other promising approaches for analyzing dyadic data as, for example, specific types of time series analyses (e.g., multilevel bivariate autoregressive models, see Beebe et al., 2016) or other data-analytical strategies that capitalize on the time-structuredness of data on different time scales (e.g., Xu et al., 2020).

Overall, research based on the lifeworld approach or other dynamic developmental systems theories should strive for converging evidence, namely that findings from different samples and methodological approaches are pointing to the same conclusions. Equipped with, first, a conceptual framework grounded in a modern view of developmental processes and, second, a suite of methodological approaches, the field of developmental science is ready to set out to explore the complexities of child development in a way that will enable developmental science to better describe, explain, and modify the fundamental processes of human development. In particular, the lifeworld approach calls for a paradigm shift in the way of thinking about social-cognitive development in that the focus on the individual child must be extended and complemented by not only assuming, but by providing empirical evidence for the specific and changing interactional mechanisms within the dynamic developmental system that drive social-cognitive development.

## Data availability statement

The original contributions presented in the study are included in the article/supplementary material, further inquiries can be directed to the corresponding author.

## Author contributions

JK: Writing – review & editing, Writing – original draft, Visualization, Conceptualization. MK: Writing – review & editing, Visualization, Conceptualization.
